# A Novel Multiplex LAMP Assay for the Rapid and Accurate Diagnosis of Visceral Leishmaniasis Caused by *Leishmania infantum* from Iran

**DOI:** 10.1155/2023/9326183

**Published:** 2023-11-18

**Authors:** Sahar Samsami, Sahar Namavari, Saeed Ataei, Abdolmajid Ghasemian, Ava Yazdanpanah, Neda Sepahi, Gholamreza Hatam, Hossein Faramarzi, Hadi Mirzaei, Razie Ranjbar, Ali Ghanbariasad

**Affiliations:** ^1^Student Research Committee, Fasa University of Medical Sciences, Fasa, Iran; ^2^Department of Medical Biotechnologies, Fasa University of Medical Sciences, Fasa, Iran; ^3^Noncommunicable Diseases Research Center, Fasa University of Medical Sciences, Fasa, Iran; ^4^Department of Medical Parasitology and Mycology, School of Medicine, Shiraz University of Medical Sciences, Shiraz, Iran; ^5^Department of Community Medicine, Shiraz University of Medical Sciences, Shiraz, Iran; ^6^Department of Medical Genetics, School of Medicine, Zabol University of Medical Sciences, Zabol, Iran

## Abstract

Visceral leishmaniosis (VL) is one of the neglected tropical diseases despite being responsible for serious clinical symptoms, some of which lead to fatal outcomes. Thus, there is a need to apply accurate, rapid, and specific diagnostic measurements in order to control the disease and reduce the mortality rate. We aimed to develop and validate a multiplex LAMP assay for the diagnosis of VL caused by *Leishmania infantum* (*L. infantum*). Moreover, a thorough assessment was conducted to determine the effectiveness of multiplex LAMP in identifying various *Leishmania* species, such as *Leishmania tropica* (*L. tropica*) and *Leishmania major* (*L. major*) in comparison to *Leishmania infantum* (*L. infantum*). The diagnostic performance of the multiplex LAMP method for VL was compared to each LAMP assay, real-time polymerase chain reaction (RT-qPCR), and nested PCR technique. Two separated primers were set and used in a multiplex LAMP assay which was designed based on the ITS2 (internal transcribed spacer II) and were selected on the basis of conserved and high copy number region. Multiplex LAMP primers were designed using an online tool available at https://www.primerexplorer.jp/e. The alignment was performed using MEGA5, and the primers were further adjusted utilizing GENE Runner software. All molecular methods were tested on the serial dilution of cloned plasmid containing ITS region from standard strains of *L. infantum*, *L. tropica*, and *L. major*. Moreover, multiplex LAMP assay was evaluated and compared based on both standard strains and 55 clinical samples from humans as well as dogs. Various approaches were applied to interpret the multiplex LAMP reaction which deciphered a higher sensitivity when compared to the RT-qPCR for *L. infantum* (one copy number of plasmid, equal to 0.85 femtograms (fg) of plasmid concentration, and 0.004 parasite DNA per *μ*L) detection while these three standard strains of *Leishmania* were confirmed to contain 40 DNA copies using RT-qPCR. Additionally, the multiplex LAMP detection limit was approximately equivalent to RT-qPCR for *L. major* and *L. tropica*, which included 0.342 picograms (pg) and 342 femtograms (fg) of plasmid concentration, 4 × 10^3^ and 4 × 10^2^ copy number of plasmid, and 17.1 and 1.71 parasite DNA per *μ*L for *L. major* and *L. tropica*, respectively. Nested PCR exhibited a lower detection limit for *L. infantum* of 4 × 10^6^ plasmid copy number compared to multiplex LAMP and RT-qPCR. Multiplex LAMP has the potential for accurate and rapid detection of infectious disease, successful treatment, and finding and monitoring asymptomatic cases, especially in low-income countries.

## 1. Introduction

*Leishmania infantum* (*L. infantum*) is a eukaryotic protozoan parasite causing visceral leishmaniasis (VL), also known as kala-azar which is transmitted by the phlebotomine sandflies. VL is the most serious and lethal form of the disease affecting internal organs such as the spleen, liver, bone marrow, and lymph nodes [1–3]. Leishmaniasis is present in 98 countries and leads to 700,000 to 1 million new cases of cutaneous leishmaniasis (CL) and 50,000 to 90,000 new cases of the more severe VL annually. If left untreated, VL can be fatal and cause approximately 20,000 to 30,000 deaths each year. A recent systematic review and meta-analysis of studies revealed that the prevalence of human VL in Iran was 0.5%. Higher rates were observed in the northern and western regions of the country, as well as among males and children under the age of 10 [4, 5]. The disease is endemic in nearly 100 countries with a 50,000 to 90,000 annual incidence rate and up to 40,000 deaths per year, making the VL a seriously neglected tropical disease. VL is caused by *L. infantum*, *L. donovani* of the old world, or *L. chagasi* of the new world. Due to the common characteristics of symptoms associated with VL and other diseases, the disease diagnosis is intricate and time-consuming [6, 7].

*L. infantum* causes millions of human deaths or reservoir cases, hence early and reliable diagnosis of the VL is crucial for case management, control and surveillance of the disease, initiation of therapy, and reduction of the disease incidence within endemic areas [2, 6, 8]. There is a need for accurate and rapid diagnosis of VL. Currently, available approaches include parasitological, serological, and molecular methods [9, 10]. Those common diagnostic techniques include *in vitro* cultivation and detection of the parasites in tissue aspirates which are mostly used for VL identification. However, these tests are time-consuming and labor working, with variable sensitivity and specificity values. These carry the potential risk of fatal hemorrhage and require a high level of expertise [8, 11]. Serological diagnostic methods such as indirect immunofluorescence antibody test (IFAT), enzyme-linked immunosorbent assay (ELISA), recombinant antigen-based immunochromatography test (ICT), and western blotting are moderately sensitive [2, 3, 12]. These techniques may give false-negative results and cross-reactions with other diseases and lack the ability to determine the current or previous infection [12].

More recently, polymerase chain reaction (PCR)-based methods have been applied for rapid and sensitive detection, identification, and quantification of various *Leishmania* spp. [11–13]. Notwithstanding conventional PCR, nested PCR, or real-time PCR (RT-qPCR) targeting the *Leishmania* spp. genome and using different primer pairs for amplification, the RT-qPCR has appeared as a highly sensitive technique for the VL diagnosis [12, 14]. Nevertheless, sensitivity and specificity of these assays depend upon different aspects such as the DNA extraction method, clinical material, primers which target copy numbers, and technical conditions. Moreover, the need for sophisticated expensive equipment along with technical expertise and high costs has made these assays unaffordable/unavailable in deprived (low-income) regions [3, 12].

The loop-mediated isothermal amplification (LAMP) has been successfully applied as a rapid, sensitive, and highly efficient technique compared to the conventional PCR technique in field-based situations [3, 9, 15]. It is performed at isothermal amplification due to strand displacement activity of *Bacillus stearothermophilus* (*Bst*) DNA polymerases. High specificity of this method is due to the use of several separate primers recognizing six (or eight) distinct regions on the target gene. Accordingly, 10^9^-10^10^ copy numbers of target gene were amplified in an hour under constant temperature (60–65°C) [1, 16]. These primer sets include two outer (F3 and B3) primers, two inner primers (forward inner primer (FIP) and backward inner primer (BIP)), and loop primers [17]. Pyrophosphate ions generated in the LAMP reaction bind to manganese ions, thus facilitating the visualization of calcein and products using green fluorescence (SYBR Green I), turbidity (spectrophotometry measurement of absorbance at 400 nm), and agarose gel electrophoresis. Because of the inhibitory and false-positive aspects of dyes, these must be added to postreaction conditions [10, 18].

Multiplex LAMP was applied to the LAMP reaction because the progression of the method could potentially increase the sensitivity of the test, which means the ability to detect low levels of parasite DNA [17, 19]. To target genes in *Leishmania* spp., kinetoplastid DNA (k-DNA), small subunit rRNA (SSU rRNA), and ribosomal internal transcribed spacer (ITS) regions are usually used. ITS region is a noncoding spacer DNA placed between the 18s rRNA and 5.8s rRNA with 40 to 200 copy numbers [18, 20]. Theoretically, we expect that multiplex LAMP produces an overwhelming amount of DNA products, a feature that can detect even minor template DNA amounts in samples. Eventually, the sensitivity would be enhanced. Herein, we aimed to develop and validate a novel multiplex LAMP assay for the rapid and accurate diagnosis of *L. infantum*. Two sets of primers were selected for multiplex LAMP to target a single gene region in order to enhance the detection limit and sensitivity of clinical sample diagnosis. Each set of primers is capable of detecting six distinct regions, and the strand displacement feature allows for the replication of a large amount of DNA that can be diagnosed using turbidity and SYBR Green I. Additionally, the accuracy of using the ITS2 region as a target DNA is being evaluated for the first time on both standard strains and clinical samples. The ITS2 region, also known as “internal transcribed spacer 2,” is a segment of the rRNA gene found in the Leishmania parasite. Due to its high variability across different Leishmania species, it is a valuable tool for species identification. The ITS2 region has a length of approximately 50–650 base pairs and a species-specific copy number in Leishmania [21–23]. Also, this technique is compared with other molecular techniques, such as RT-qPCR and nested PCR, as well as simple LAMP assays to identify L. infantum that causes VL.

## 2. Materials and Methods

A schematic diagram of this study has been provided in this study as a graphical abstract.

### 2.1. Ethics Statement

The study was approved and carried out under the guidelines of the ethical review committee of Fasa University of medical science (ethical code: IR.FUMS.REC.1395.130). Consent for the inclusion of young children was obtained from parents or guardians. Additionally, ethics approval granted the permission to collect dog samples used in this study and the sampling consent of dog keepers was taken.

### 2.2. Sample Preparation and DNA Extraction

The reference strains of *L. major* (MHOM/IR/75/ER), *L. infantum* (MCAN/IR/97/LON490), and *L. tropica* (MHOM/SU/74/K27) promastigotes were cultured in Gibco RPMI1640 Medium (Thermo Fisher Scientific, US) supplemented with 10% heat-inactivated fetal bovine serum (FBS) (Thermo Fisher Scientific, US) and 100 IU of penicillin with 100 *μ*g/mL of streptomycin (Gibco, Thermo Fisher Scientific, US) and stored at 25°C shaker incubator (TiaTech-BR-12SH). The parasites cultivated were harvested in the late log phase and then were washed three times using phosphate-buffered saline (PBS) [24]. DNA was extracted using the QIAMP Mini Kit (Qiagen, Hilden, Germany) following the manufacturer's protocol. Finally, the density was approximated using a NanoDrop spectrophotometer (Biotech, Synergy HTX) and concentration was adjusted to 34.2 ng/*μ*L. Next, the DNA samples were kept at −20°C for further analysis.

### 2.3. Clinical Samples

Among suspected cases (*n* = 105), clinical samples consisting of symptomatic and asymptomatic cases were collected from southern Iran between the summer of 2019 and winter of 2020 and had previously undergone molecular testing, biopsies, and blood smear. Venous blood samples (5 mL) were taken from 15 dogs and 40 patients with the pretreatment stage of VL. Also, 50 blood samples of asymptomatic individuals from nonendemic regions were used as negative controls. All of the VL cases were *L. infantum.* The verification of positive samples was based on the clinical symptoms, positive PCR products, and blood smear or biopsy from the spleen or bone marrow (dog's samples were biopsy from both spleen and bone marrow and human obtained from spleen biopsy). The whole blood samples were centrifuged at 900 × g for 10 min to separate the buffy coat, and DNA was extracted from 300 *μ*L of each buffy coat using the QIAMP Blood Mini Kit (Qiagen, Hilden, Germany) according to the manufacture's protocol. The quality of DNA was measured using a NanoDrop spectrophotometer (BioTek, Synergy HTX, and USA) at 260/280 and 260/230 ratios and were eluted to 30 *μ*L final volumes and then stored at −20°C for further analysis. Moreover, fifteen DNA samples were extracted from canine VL or CVL dogs which were confirmed and obtained by the Shiraz parasitology department, Shiraz University of Medical Sciences.

### 2.4. Negative Control

Genomic DNAs from *Trypanosoma cruzi*, *Plasmodium vivax*, and *Escherichia coli* were used as negative samples provided by Dr. Hatam in the parasitology department of Shiraz University of Medical Sciences. Furthermore, 50 samples were collected from healthy individuals living in a nonendemic region.

### 2.5. TA Cloning

Primer design for PCR cloning based on the ITS2 region was conducted using primer BLAST from NCBI online software and was confirmed by online primer3 of the NCBI (Table 1).

PCR conditions are exhibited in Table 2.

Positive PCR products were purified and sent to U2Biol Co., Ltd., South Korea, for sequencing. Sequences were confirmed as standard strain by Bio Edit Version 7.0.1 and carried out to validate multiple alignments with reference to *Leishmania* strains found from the Genebank. TA cloning was accomplished by Thermo Scientific Kit (Thermo Fisher, USA) and *E. coli* TOP10 was used as host and Ptz57R plasmid was used as a vector. Blue-white screening was done and confirmation of TA cloning was performed by colony PCR. Plasmid extraction was carried out using alkaline-lysis protocol [25].

### 2.6. Nested PCR

Nested primers were designed by Gene Runner and checked by mega 5 and primer3 online software of NCBI (Table 3).

The reaction mixture included 20 *μ*L of the total volume containing 10 *μ*L of 2X master mix (Amplicon Red, Denmark), 1 *μ*L of each 10 *μ*M forward and reverse primers, ddH_2_O, and 3 *μ*L of 50 ng/*μ*L DNA template. The amplification was conducted in the thermal cycler (BioradT100, USA) with the conditions of 95°C for 5 min and 30 cycles of the following steps: denaturation at 95°C for 30 secs, annealing at 54.5°C for 20 secs, and extension at 72°C for 30 secs. After the completion of 30 cycles, the reaction was subjected to the final extension at 72°C for 10 min. The next nested PCR step was performed under the same condition with the same reaction mixture, except that in this case, the DNA template was 1 *μ*L of the first nested PCR product. The second step products were electrophoresed on 2% gel agarose. For equivalence, the molecular techniques for *Leishmania* spp. detection were carried out on serial dilution of the extracted plasmid.

### 2.7. Real-Time PCR

The ITS2 sequence obtained from NCBI and region-specific primers were designed using Allele ID software and checked by primer3 of NCBI based on the ITS2 region (Table 4).

The reaction mixture consisted of 7.5 *μ*L of High ROX Red master mix (Amplicon, Denmark), 0.5 *μ*L of 10 *μ*M forward and reverse primers, and 1 *μ*L of DNA template. The program (absent-present) was performed on the ABI peal-time PCR, Sep one plus system, under the following conditions: initial denaturation at 95°C for 3 min, denaturation step at 95°C for 45 secs, annealing at 55°C for 45 s, extension at 72°C for 60 s, and final extension at 72°C for 10 min.

### 2.8. LAMP and Multiplex LAMP

Two different sets of LAMP primers (external primers: F3 and B3; internal primers: FIP and BIP) were designed as multiplex based on the ITS2 sequence of *Leishmania* species under the names LAMP1 and LAMP2. Two sets of primers were designed by Primer Explorer V5 online software (https://www.primerexplorer.jp/e). The consensus sequences of various Leishmania species from NBI were aligned using MEGA5 software, along with our standard strains that were sequenced. We evaluated five sets of primers proposed by Primer Explorer online tools and selected two sets based on specific criteria. These criteria included their ability to detect our standard strain of *L. infantum* and other *Leishmania infantum* sequences from NCBI, nucleotide distances between primers, recognition of six distinct regions of the ITS region, and melting temperatures between 65°C and 75°C, replicating the whole selected region of DNA target. After the evaluation of 5 proposed sets, 2 of them met our criteria which were selected for further evaluation. With regard to avoiding self-complementarity, hairpin formation, primer-dimer formation, and GC content with the 3′ of a primer ending in G or C, GENE Runner software was used and finally these 2 sets of primers were blasted using NCBI primer blast, and the result revealed that they are specific primers for *L. infantum* detection (Table 5).

The components of the LAMP reaction mixture included 1 *μ*L of primer mix (5 pmol of each F3, B3, and 40 pmol of each FIP and BIP), 1 *μ*L (8 U) of Bst DNA polymerase, 2 *μ*L of DNA sample, 12.5 *μ*L of 2x LAMP buffer containing 1.4 mM of each dNTP, 0.8 M betaine, 20 mM Tris-HCl (pH 8.8), 10 mM KCl, 10 mM (NH4)_2_SO_4_, 8 mM MgSO_4_, and 0.1% Tween 20. To find the ideal amplification conditions, the mixture was examined with both primer sets, and the reactions were carried out at temperatures ranging from 59°C to 65°C for incubation durations of 120 min in a water bath to optimize amplification and facilitate detection.

The multiplex LAMP reaction was performed in 25 *μ*L total volume reaction mixture which contained 2.5 *μ*L of enzyme reaction buffer (New England Biolabs Inc., MA, USA), 1 *μ*L (8U) of *Bst* DNA polymerase (New England Biolabs Inc., MA, USA), 1 *μ*L of a DNA template, 8.5 *μ*L mixture volume of 1.4 mM of dNTPs, 0.8 M Betaine, 8 mM MgSO4 and 4.5 *μ*L of primer mix of LAMP1, and 2 sets (5 pmol of each F3, B3 and 40 pmol of each FIP and BIP). In order to establish the optimum condition of the multiplex LAMP, several assays were examined in temperatures and time ranging from 59°C to 65°C for 60 to 120 min, respectively. Finally, a multiplex LAMP reaction was performed at 60°C for 2 hrs, incubated in the water bath for optimal detection of a lower concentration of the amplified product.

### 2.9. LAMP and Multiplex LAMP Visual Detection

At the end of incubation time, SYBR Green I (Thermo Fisher Scientific, Grand Island, NY) diluted in 1 : 10 ratio was added to amplified products, and an immediate color change from pink to green indicated positive result.

### 2.10. Detection Limit

**Figure 1:**
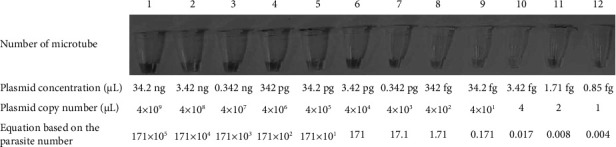
Serial dilution of extracted plasmid and the property of each tube.

Minimum or limit of detection of molecular tests was estimated using 10 folded-plasmid serial dilutions (*n* = 12) of *L. infantum*, *L. tropica*, and *L. major* which had DNA concentrations ranging from the 4 × 10^9^ (34.2 ng/*μ*L) to 1 copy number/*μ*L (0.85 fg/*μ*L) using copy number to plasmid online website calculator which obtains minimum detection thresholds (Figure 1). A negative control sample containing distilled water was also used.

### 2.11. Clinical Samples Analysis

After the DNA extraction from samples of VL cases given positive results by PCR, smear, and clinical symptoms, the multiplex LAMP technique was used to investigate 55 samples according to the previous conditions. The results were further analyzed when test tubes were supplemented with CYBER green I. For comparison, PCR, real-time PCR, and LAMP methods were performed on all clinical samples.

### 2.12. Statistical Analysis

After evaluation of clinical samples with different molecular methods, we used the following formula to calculate sensitivity and specificity. The sensitivity = number of true positives/(number of true positives + number of false negatives). Specificity = number of true negative/(number of false positive + number of true negative).

## 3. Results

### 3.1. Nested and Real-Time PCR

The results presented that all the negative controls and negative samples were negative. Nested and RT-qPCR were examined on the 12-fold serial dilutions of cloned plasmids which contained the ITS2 region of *L. infantum, L. major,* and *L. tropica* strains.

**Figure 2:**
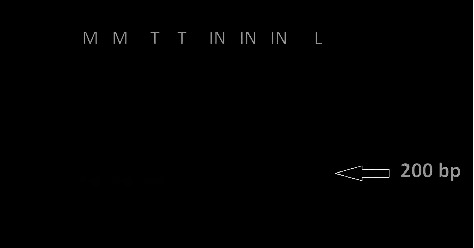
Nested PCR of *L*. *major* (M), *L. tropica* (T), and *L. infantum* (IN) on the left side of the ladder (L) were loaded in 2% gel agarose electrophoresis. Nested PCR detected 4 × 10^6^ plasmid copy number/*μ*L of *L. infantum* and *L. tropica* and detected 4 × 10^5^ plasmid copy number/*μ*L of *L. major*.

The copy number threshold for strains in the nested PCR included 4 × 10^6^, plasmid copy number or 34.2 pg of plasmid concentration equal to 171 × 10^2^ number of parasite per µL (tube number 4) for both *L. infantum* and *L. tropica,* and 4 × 10^7^ DNA plasmid copy number equal to 0.342 ng plasmid concentration or 171 × 10^3^ number of parasites per *µ*L (tube number 3) of *L. major*, which were loaded in 2% gel agarose for electrophoresis to visualize the result (Figure 2).

**Figure 3:**
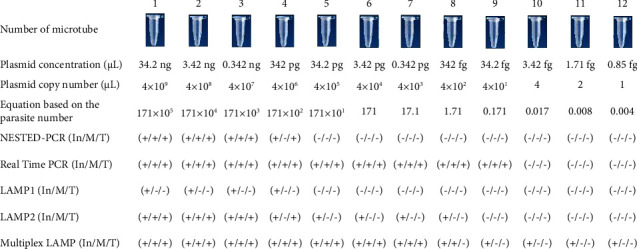
Properties of serial dilution microtubes of plasmid cloned with ITS2 region; Overall multiplex LAMP and real-time PCR were more sensitive than nested PCR for *L. infantum*, *L. major*, and *L. tropica*. On the account of *L. major* and *L. tropica*, multiplex LAMP, and real-time PCR depicted almost equal sensitivity but about *L. infantum*, multiplex LAMP was more sensitive than real-time PCR.

The study determined the copy number threshold for various strains using RT-qPCR. The detection limit was found to be 4 × 10^1^ plasmid copy number for *L. infantum*, *L. tropica*, and *L. major* which corresponds to 3.42 fg of plasmid concentration, equivalent to 0.171 number of parasites per *µ*L (tube number 9). These results demonstrated that the RT-qPCR detection limit is 40 DNA copy numbers of cloned plasmid for all the *Leishmania* strains (Figure 3).

### 3.2. Simple LAMP

**Figure 4:**
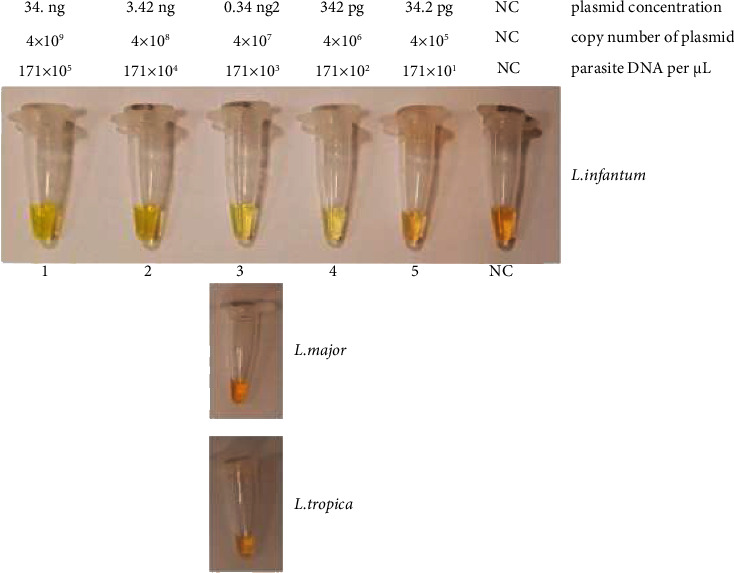
The result of the first set of LAMP primers (LAMP1) on *L. infantum*, *L. major,* and *L. tropica* extracted plasmid serial dilution. NC: negative control. This set could not identify *L. major* and *L. tropica*.

**Figure 5:**
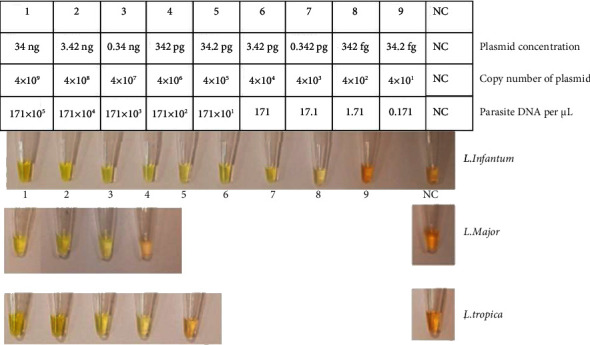
The result of the second set of LAMP primers (LAMP2) on *L. infantum, L major,* and *L. tropica* extracted plasmid serial dilution with each tube number feature; NC: negative control.

Two sets of LAMP primers were applied on the 12-fold serial diluted plasmids of three standard strains. The results depicted that the detection limit of the first set of LAMP primer (LAMP1) for *L. infantum* was 0.342 ng plasmid concentration, 4 × 10^6^ copy number of plasmid, or 171 × 10^2^ number of the parasite DNA per *μ*L (tube numbers 1–4 which have a stream of green color and tube number 5 was orange that means negative). However, the LAMP primer sets were not able to identify the other two species (*L. major* and *L. tropica*); tube number 1 was orange in color for both of them (Figure 4). This particular set can be used specifically for the detection of *L. infantum* but with a lower detection limit of 0.342 ng. The **s**econd set of primers (LAMP2) was used in a simple LAMP condition like LAMP1 set. Results of the LAMP2 test on *L. infantum*, *L. major*, and *L. tropica* included 34.2 ng, 0.342 ng, and 0.0342 ng plasmid concentration or 4 × 10^2^, 4 × 10^6^, and 4 × 10^5^ plasmid copy numbers per *μ*L or tube numbers 8, 3, and 4 (Figure 5). In comparison to RT-qPCR, the LAM2 primers have a lower detection limit. Upon evaluation using MEGA5 software and BLAST with NCBI, it was discovered that the B3 primer of the LAMP2 primers can detect a region that is conserved in *L. infantum* without any mismatch and can also detect *L. major* and *L. tropica* with a mismatch. This finding led us to consider evaluating these two sets as multiplex LAMP.

### 3.3. Multiplex LAMP

**Figure 6:**
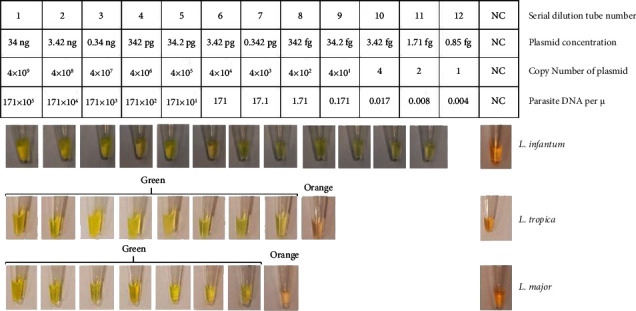
Multiplex LAMP test on standard strains of *L. infantum*, *L. tropica*, and *L. major* in sequence extracted plasmid serial dilution with each tube number feature. The green district is positive and the orange district which is the last tube is negative.

The detection limit of the multiplex LAMP assay was assessed by performing on the 12-fold serial diluted plasmids of *L. infantum, L. major, and L. tropica*. The results indicated that the detection limit of the multiplex LAMP for *L. infantum* was one copy number of plasmid, 0.85 fg of plasmid concentration, and 0.004 parasite DNA per *μ*L or tube number 12 (each 200 fg of DNA plasmid is equal to one parasite based on the website calculator (https://cels.uri.edu/gsc/cndna.html), while detection limit of RT-qPCR was 40 DNA copy numbers for all the *Leishmania* strains (tube number 8). It can be concluded that multiplex LAMP is more accurate than RT-qPCR for *L. infantum* detection. Furthermore, the detection limits of the multiplex LAMP for L. major and L. tropica were 0.342 pg, 342 fg plasmid concentration, 4 × 103, 4 × 102 copy number of plasmid and 17.1, 1.71 parasite DNA per *μ*L, respectively (Figure 6).

To confirm the positive results of the multiplex LAMP, the negative control without DNA was loaded on 1.5% gel agarose electrophoresis.

Clinical sample evaluation: the diagnostic sensitivity of multiplex LAMP assay and other molecular tests was investigated by examining 105 clinical samples, 50 of which were verified as negative and 55 as positive samples. The RT-qPCR, nested PCR, and two conventional LAMP tests, in which those primers were utilized simultaneously as multiplex LAMP, were performed to test 55 positive and 50 negative clinical samples. Notably, 35 and 50 samples were positive using the LAMP1 and LAMP2 assays, respectively. When two sets of primers were used in a multiplex LAMP test, all 55 positive samples were accurately identified as positive. RT-qPCR and nested PCR assays were also tested on clinical samples in which 48 and 45 out of 55 positive samples were identified as positive in sequence for VL caused by *L. infantum* (Table 6). It is noticeable that 55 clinical samples were also investigated by the multiplex LAMP method and all of them were positive for the VL agent (*L. infantum*) (Table 6).

## 4. Discussion

Leishmaniasis is a substantially neglected tropical disease needing profound accuracy of diagnosis, particularly in the case of the VL [26]. Multiplex LAMP is a recent development that enables the detection of multiple target sequences simultaneously. This has the potential to improve diagnostic accuracy and reduce the risk of misdiagnosis, particularly in underdeveloped and developing countries where neglected tropical diseases like leishmaniasis are prevalent [27]. The LAMP assay system is an affordable molecular diagnostic platform for leishmaniasis, with a disruptive cost advantage of over 10 times compared to imported devices with similar capabilities [28]. LAMP has been utilized for detecting various parasitic infections including malaria, Chagas disease, and schistosomiasis, in addition to leishmaniasis [29]. The modification of the LAMP reaction conditions and ingredients has improved and increased the sensitivity of the diagnostic test. The design of two primer sets for a region and simultaneous use of them in a reaction enhanced the detection range of *L. infantum* at 0.85 fg containing one plasmid, while in the best conditions and the best set amongst the primers herein, the detection range for *L. infantum* was 342 fg of DNA (4 × 10^2^ copies of the plasmid). These results demonstrated that the multiplex LAMP had a higher detection limit for *L. infantum* identification than those for the *L. tropica* and *L. major*. In a study conducted by Karani et al., two sets of primers were designed in which the sensitivity of primer set 1 varied depending on the *Leishmania* species tested, with a detection limit ranging from 30 pg to 3.6 fg. Meanwhile, primer set 2 exhibited high sensitivity, although its sensitivity also varied depending on the *Leishmania* species tested [7]. In the current investigation similar to that of Karani et al., it was found that the LAMP2 (set 2 of LAMP primers) showed greater sensitivity of 342 fg than LAMP1 (set 1 of LAMP primers) and 342 pg for detecting *L. infantum*, but not for the other two standard strains (*L. major* and *L. tropica*). This finding led to the decision to conduct a multiplex LAMP test for improved accuracy of *L. infantum* detection.

Leishmaniasis is a substantial neglected tropical disease needing profound accuracy of diagnosis, particularly in the case of the VL [26]. Recently, RT-qPCR was introduced as the gold standard of *Leishmania* spp. diagnosis, in order to compare molecular tests and further investigation toward sensitivity of molecular diagnostic approaches. Among various molecular methods for the diagnosis of leishmaniasis, RT-qPCR has exhibited high level of efficiency, not only for detecting or measuring parasite load but also as a practical tool in species detection as well as epidemiological studies. A review study of Galluzzi et al. in 2018 introduced the RT-qPCR as a sensitive and specific method for the diagnosis of leishmaniasis. However, due to the high costs of equipment posed by countries, the RT-qPCR was not considered a routine diagnostic method [30]. In a study by Dixit et al., among 267 participants (197 VL and 88 controls), 98.32% and 96.59% of sensitivity and specificity, respectively, were observed using LAMP assay in the disease diagnosis. The LAMP assay was significantly more highly efficient than the rK39 antigen rapid diagnostic test (RDT) method [2]. RT-qPCR and nested PCR were compared in the present study, in which both techniques were applied on standard serial diluted samples from standard strains of *L. infantum*, *L. major*, and *L. tropica* based on the same sequence (ITS2). It was revealed that RT-qPCR was more efficient than nested PCR (∼34.2 fg of plasmid/*μ*L). This is when multiplex LAMP exhibited more efficiency than RT-qPCR for *L. infantum* identification in the current study, multiplex LAMP with 0.85 fg detection limit, and RT-qPCR with 34.2 fg, while in another study, multiplex LAMP assays exhibit comparable diagnostic accuracy to conventional RT-qPCR, with high sensitivity and specificity values [31].

One study by Abbasi et al. in 2016 deciphered a sufficient limit of detection using the LAMP method detecting approximately 0.1 pg of the *Leishmania* spp. DNA. Indeed, the LAMP test was about 10^4^ times more sensitive than ITS-based PCR and approximately 10^3^ times more sensitive than k-DNA PCR [1]. Another LAMP test was performed on *L. tropica*, *L. donovani*, and *L. major* which reported a detection limit of 1 fg per reaction. The reaction duration was approximately 30 minutes and the diagnostic range was around 25 copies per reaction. Verma et al. exhibited that the LAMP test was able to detect 64 positive blood samples out of 66 cases, with a sensitivity of 96.9% and 65 out of 67 skin samples of VL cases with a sensitivity of 97% and 8 out of 10 skin biopsy specimens with a sensitivity of 80%. The LAMP was also introduced as a powerful and reliable tool for rapid and effective detection of VL and CL. One of the reasons for this is due to the use of four separate primers amplifying six regions of the target gene simultaneously. In addition to the primers, for the final identification of the products, they used SYBR Green I [3]. In our experiment, likewise, Verma et al. designed primers for multiplex LAMP reaction which were more efficient for *L. infantum* identification than *L. tropica* and *L. major*. The cause can be related to the difference in the sequence or copy number of the target gene in these two strains [3]. It is observed that the LAMP assay was a more sensitive tool for CL detection than alternative diagnostic methods, also being more sensitive than the gold standard method when considering using minimal invasive sampling. DNA along with 18S rDNA has been used in their study when it comes to template targets [25]. Also, Jang et al. in 2021 proved that the multiplex LAMP assay results were comparable to those of RT-qPCR for the diagnosis of SARS-CoV-2 [32].

In 2018, Mukhtar et al. designed a VL diagnosis kit based on the multiplex LAMP method. The sensitivity of this kit was 97.6% and its specificity was 99.1%. The method contained two sets of primers which target two distinct regions (DNA k, 18s rRNA). The reaction mixture incubated for 40 minutes at 65°C and the final products were observed under an LED light. The result showed that the multiplex LAMP is just as capable as the applied method for designing detection kits [15]. On the other hand, Karani et al. designed two sets of multiplex LAMP reaction primers based on the 18s rRNA region which was used to diagnose CL, VL, and mucosal leishmaniasis. The detection limit of this test ranged from 30 pg to 3.6 fg, depending on the leishmaniasis strains. The use of two sets of primers improved the detection limit. Another reason for designing a multiplex LAMP based on a gene region was the detection of a wide range of *Leishmania* spp. using two sets of primers [7].

In another study, the LAMP assay sensitivity and specificity included 95% and 86%, respectively, for the diagnosis of the CL and 92% and 100%, respectively, for the VL in the whole blood. It was proposed that the LAMP assay was reliable in noninvasive sampling for the leishmaniasis [26]. The portable LAMP device could detect 100 fg of *L. donovani* DNA in clinical samples having 100% and 98% sensitivity and specificity, respectively [28]. A systematic review and meta-analysis in 2022 revealed that the LAMP assay had significant higher efficacy than conventional and PCR methods in blood samples for the VL diagnosis (estimate values of > 90% and area under the curve (AUC) values > 0.96) [33].

The present study introduced data on a developed LAMP method called multiplex LAMP as a detection kit capable of diagnosing *L. infantum* with a higher level of detection than those of *L. major* and *L. tropica*. The ITS region was adopted as the target gene characterized by a high copy number and conservation among the *Leishmania* species [30]. Two primer sets were multiplexed to ensure the enhancement of the VL detection range accompanied by the ability to detect other species (*L. major* and *L. tropica*). The composition of the primers was appropriately performed on serial dilution to ensure a reliable and robust new multiplex technique. A highly sensitive and specific multiplex LAMP represented a detection limit of 0.004 parasites per *μ*L for *L. infantum* as well as, approximately, 17.1 and 1.71 parasites per *μ*L for *L. major* and *L. tropica*, respectively, on the plasmid serial dilutions. In this study, the diagnostic sensitivity of multiplex LAMP assay and other molecular tests was investigated by examining 105 clinical samples, of which 50 cases were verified as negative and 55 as positive. All clinical samples were confirmed using PCR, various existing clinical signs, and a biopsy smear. The RT-qPCR, nested PCR, and two conventional LAMP tests, in which those primers were utilized simultaneously as multiplex LAMP, were performed to test 55 positive and 50 negative clinical samples. In any individual LAMP assay, 35 samples were positive by the LAMP1 test and 50 of them were positive by the LAMP2 test. When two sets of primers were used in a multiplex LAMP test, all 55 positive samples were accurately identified as positive. LAMP1 and LAMP2 have a sensitivity of 63% and 100% and a specificity of 90% and 98%, respectively. This is when the combined sensitivity of multiplex LAMP was 100% and its specificity was 96%. Although the specificity decreased at an inappreciable amount of approximately 2%, the sensitivity enhanced when the combination of two sets of primers LAMP 1 and 2 was used in a single reaction. Bioinformatics analysis using online tools for primer evaluation showed that this increase in sensitivity may be due to the reinforcement effect of the B3 primer of LAMP2. In addition to this, the multiplex LAMP assay uses several primer sets that target distinct regions of the *Leishmania* genome, thereby improving the sensitivity and specificity of the assay. This approach to multiplexing enables the identification of multiple *Leishmania* species or strains simultaneously, facilitating differential diagnosis and accurate identification of the infecting species and leprosy infection [34]. Using two sets of primers simultaneously, one of them (LAMP1) specifically recognizes *L. infantum* and the other one (LAMP2) detects *L. infantum* with a higher detection limit. This may significantly increase the sensitivity of the assay for the diagnosis of leishmaniasis, particularly of VL. This improved accuracy assists in preventing false-negative results, which might lead to failure of the treatment and lead to the maintenance of reservoirs [30]. This enhanced detection limit enables the identification of minimal amounts of pathogens, even in viral infections like COVID-19 with samples demonstrating elevated Ct values [35]. RT-qPCR and nested PCR demonstrated similar patterns to each other with 87% and 81% sensitivity and 100% specificity sequentially. Moreover, both of them followed the homological template of clinical sample detection. In other words, RT-qPCR in spite of rapidity and accuracy in the diagnosis, it is challenging in subclinical cases, while LAMP assay can be applied for the detection of *Leishmania* spp. infections in both humans and reservoir animals [7]. Clinical samples were collected and tested via the multiplex LAMP, which proved to successfully identify all the specimens [34]. There is a scarcity of studies focusing on the development of a multiplex LAMP based on a single region of the gene to improve not only the detection limit but also sensitivity of clinical sample diagnosis. Furthermore, this is almost the first time that the ITS2 region was considered as a target gene and presented acceptable accuracy based on the clinical sample results. Also, this kind of multiplex LAMP accurately detected all the infected clinical samples, whereas RT-qPCR and nested PCR were not able to detect all of them considering the same conditions. This finding strongly supports the potential of multiplex LAMP assay for the identification of *L. infantum* causing VL. Effective primer design is crucial for the success of LAMP assays. It is essential for the primers to be specific in order to avoid cross-reactivity with other organisms or genes [10]. The use of multiple primers in a multiplex LAMP assay may increase the risk of nonspecific binding and false positives [36]. In addition, we encountered several challenges during our multiplex study, such as accurately setting the concentration of primers, preventing contamination, and constantly monitoring the test with negative and internal controls. We also need to have confidence in our cloning and sequencing processes to ensure accuracy. Additionally, we had to carefully collect samples from untreated patients.

Although there are some limitations and difficulties, LAMP has proven to be a fast, specific, and effective method for detecting *Leishmania* infection. The development of portable LAMP devices will open new avenues for diagnosing and predicting outcomes of *Leishmania* infections at the point of care in the future. This portable device has the capability to detect and amplify even very small amounts of *L. donovani* DNA, as low as 100 fg. The LAMP detection system is equipped with unique features, such as a compact fluorescent detection device, which can be utilized to measure the intensity of fluorescence produced by LAMP products [28]. By utilizing self-quenching and dequenching fluorogenic probes, the LAMP system could become even more versatile, leading to the development of a reliable real-time multiplex LAMP for point-of-care setting [10].

## 5. Conclusion

Our results suggested that the multiplex LAMP method with two primer sets designed for the ITS2 region was a highly specific tool with a detection limit approximately equal to the RT-qPCR. The multiplex LAMP design has the sensitivity to detect 0.85 fg of DNA containing 1 copy of the plasmid (200 copy numbers of ITS region which are cloned in the plasmid are considered to be 1 parasite; 1 parasite has 200 copy numbers of this gene). Results for the negative samples and controls depicted a high specificity (96%), suggesting the multiplex LAMP as a promising platform available for the identification of the VL causative agent *L. infantum*.

## Figures and Tables

**Table 1:** Primer sequences for TA cloning.

Gene	TM	Primer	Sequence	Product size (bp)
ITS2	53.76	Forward	CATTTTCCGATGATTACACCCAA	977
	53.2	Reverse	TCTTTTTTCTCTGTGCGTAC	

**Table 2:** PCR amplification of the ITS2 region of Leishmania was amplified by T100 thermal cycler (Bio-Rad, USA).

Temperature (°C)	Time	Steps	
95	5 min	Early denaturation	

95	30 sec	Denaturation	Cycling steps ≠ 30
49	30 sec	Annealing	
72	90 sec	Extension	

72	10 min	Final extension	

**Table 3:** Nested external and internal primers.

Gene	TM	Primer	Sequence	Product size (bp)
ITS1, ITS2, 28S (external)	57.3	Forward	AGGCGTGTGTTTGTGTTGTG	439 bp
	58.24	Reverse	AGAGTGAGGGCGCGGATA	

ITS1, ITS2, 28S (internal)	59.35	Forward	AACTCCTCTCTGGTGCTTGC	189 bp
	55.25	Reverse	AAAATGGCCAACGCGAAGTT	

**Table 4:** Real-time PCR primers for *Leishmania* species.

Gene	TM	Primer	Sequence	Product size (bp)
ITS2	57.3	Forward	AGGCGTGTGTTTGTGTTGTG	137 bp
	59.3	Backward	GCAAGCACCAGAGAGGAGTT	

**Table 5:** Two sets of LAMP1, 2 primers designed for multiplex LAMP.

Gene	TM	Primer	Sequence
ITS2 LAMP SET1	54.66	F3	TTCTCTTTTTCTCTCTCCATTC
	52.35	B3	ACACACACAACCTACTTCT
	75.16	FIP	TCCTGGTCACAGCCTCTCTCTCCTCTCTTTTTTCATCAAAAAGG
	73.45	BIP	AACGAGAATTCAACTTCGCGTTGACACAGAGAGAGAGCCAC

ITS2 LAMP SET2	52.01	F3	ACCAAAACGAGAATTCAACTT
	53.20	B3	TCTTTTTTCTCTGTGCGTAC
	73.55	FIP	TACCACACAGAGAGAGAGCCACCGTTGGCCATTTTTTGCT
	73.25	BIP	TAGAAGTAGGTTGTGTGTGTGTATGTATGAGAGAGTGAGGGCG

**Table 6:** Results of testing molecular tools on the clinical samples.

Samples	Real-time PCR	Nested PCR	LAMP1	LAMP2	Multiplex LAMP
50 negatives	—	—	—	1 positive	2 positives
55 positives	48 positives, 7 negatives	45 positives, 10 negatives	35 positives, 20 negatives	50 positives, 5 negatives	55 positives, 0 negative
	(Sensitivity 87% and specificity 100%)	(Sensitivity 81% and specificity 100%)	(Sensitivity 63% and specificity 100%)	(Sensitivity 90% and specificity 98%)	(Sensitivity 100% and specificity 96%)

## Data Availability

No underlying data were collected or produced in this study.
